# Supplementary material for: Added Value of Next Generation Sequencing in Characterizing the Evolution of HIV-1 Drug Resistance in Kenyan Youth
Source: Viruses. 2023 Jun 22;15(7):1416. doi: 10.3390/v15071416 (PMC10383797; doi:10.3390/v15071416)
Supplement: Supplementary file 1 [file viruses-15-01416-s001.zip › viruses-2454708-supplementary.pdf]

Supplementary Materials

Details of the statistical analyses:

The bootstraps in these exploratory statistical analyses sampled the participants with replacement, to account for the 6 participants in both the short- and long-term studies and to relax the normality assumption. As many models were fit, we present the results with confidence intervals, do not affix statistical significance and treat the findings as exploratory.

Equation (1) describes the modelling approach used for both hypotheses:

$$Y_{TP2} = S + A + Y_{TP1} + Y_{TP1} \cdot A + Y_{TP1} \cdot S. \quad (1)$$

To address the first hypothesis, that both minority drug resistance variants and undetected DRMs are more likely to evolve if the DRM has positive penalty to the current regimen, we first reduce the set of all potential DRMs to those that had at least 2 observations with penalty score of 0 at the later time point and had more than 2 established (>20%) mutations at the later time point, indicating some clinically-relevant evolution.

For each DRM, we regressed the later TP NGS prevalence (logit transformed, Y_{TP2}) on the earlier TP prevalence (logit transformed, Y_{TP1}), a study indicator (S), the primary exposure (A), an interaction between earlier TP prevalence and the exposure, and an interaction between earlier TP prevalence and the study indicator (Eqn 1). The exposure was an indicator for whether at least one drug in the participant's later TP regimen had a penalty greater than 0.

We used the model to generate predicted differences in the later TP prevalence for those with penalty >0 vs. 0 among those with earlier TP prevalence (Y_{TP1}) of 0, 15% or 80% to represent absent, minority drug resistance variants, or established earlier TP prevalence. As all participants were taking NNRTI-based ART in the short-term group, and, therefore, all had penalty >0 to all NNRTI DRMs, we examined NNRTI DRMs using the long-term group only and removed the study indicator terms from the regression model.

Conversely, we did not expect mutations to evolve (from a minority drug resistance level or from 0) when there was a zero (or <0) penalty to the current regimen. To capture unexpected evolution in our dataset, we made a table with all observed mutations with a later TP penalty ≤0 that showed growth of at least 5 percentage points in mutation prevalence between TPs.

To address the hypothesis that participants with minority drug resistance variants with non-zero penalty scores to current regimens will have more impactful changes compared to those without minority drug resistance variants or with DRMs that have zero or negative penalty scores to current regimens, and along the same lines as the analyses outlined above and equation 1, we regressed the later TP Stanford resistance

scores, Y_{TP2}, on the earlier TP score, Y_{TP1}, a study indicator (S), the primary exposure (A), an interaction between the earlier TP score and the exposure, and an interaction between the earlier TP score and the study indicator. Here, the exposure was an indicator for having a minority drug resistance variant at the earlier TP with non-zero penalty. For each antiretroviral medication and study, we captured the difference in later TP Stanford score for those with at least 1 minority drug resistant variant, non-zero penalty DRM vs. those with none.

Table S1. Evolving NRTI DRMs

<table>
<tr><th colspan="5">Short-term evolution</th></tr>
<tr><td>DRM</td><td colspan="2">Regimen</td><td>TP1 NGS frequency, %</td><td>TP2 NGS frequency, %</td></tr>
<tr><td>L74V</td><td colspan="2">ABC, 3TC, NVP</td><td>none</td><td>56</td></tr>
<tr><td>Y115F</td><td colspan="2">ABC, 3TC, NVP</td><td>6</td><td>92</td></tr>
<tr><td>M184V</td><td colspan="2">ABC, 3TC, NVP</td><td>none</td><td>83</td></tr>
<tr><td>T215Y</td><td colspan="2">AZT, 3TC, NVP</td><td>none</td><td>58</td></tr>
<tr><td>T215I</td><td colspan="2">ABC, 3TC, NVP</td><td>11</td><td>43</td></tr>
<tr><th colspan="5">Long-term evolution without regimen change</th></tr>
<tr><td>DRM</td><td colspan="2">Regimen</td><td>TP1 NGS frequency, %</td><td>TP2 NGS frequency, %</td></tr>
<tr><td>L74V</td><td colspan="2">ABC, 3TC, NVP</td><td>none</td><td>99</td></tr>
<tr><td>Y115F</td><td colspan="2">ABC, 3TC, NVP</td><td>none</td><td>99</td></tr>
<tr><td>Y115F</td><td colspan="2">ABC, 3TC, NVP</td><td>none</td><td>98</td></tr>
<tr><td>Y115F</td><td colspan="2">ABC, 3TC, NVP</td><td>none</td><td>99</td></tr>
<tr><td>K219Q</td><td colspan="2">ABC, 3TC, NVP</td><td>none</td><td>81</td></tr>
<tr><th colspan="5">Long-term evolution with regimen change</th></tr>
<tr><td>DRM</td><td>TP1 regimen</td><td>TP2 regimen</td><td>TP1 NGS frequency, %</td><td>TP2 NGS frequency, %</td></tr>
<tr><td>M41L</td><td>AZT, 3TC, NVP</td><td>ABC, 3TC, LPV/r</td><td>none</td><td>80</td></tr>
<tr><td>M41L</td><td>AZT, 3TC, NVP</td><td>ABC, 3TC, LPV/r</td><td>none</td><td>72</td></tr>
<tr><td>M41L</td><td>ABC, 3TC, NVP</td><td>TDF, 3TC, ATV/r</td><td>none</td><td>100</td></tr>
<tr><td>D67N</td><td>AZT, 3TC, NVP</td><td>ABC, 3TC, LPV/r</td><td>none</td><td>99</td></tr>
<tr><td>D67N</td><td>ABC, 3TC, NVP</td><td>TDF, 3TC, ATV/r</td><td>none</td><td>100</td></tr>
<tr><td>T69D</td><td>AZT, 3TC, NVP</td><td>TDF, 3TC, NVP</td><td>none</td><td>99</td></tr>
<tr><td>K70R</td><td>AZT, 3TC, NVP</td><td>ABC, 3TC, LPV/r</td><td>none</td><td>100</td></tr>
<tr><td>K70R</td><td>ABC, 3TC, NVP</td><td>TDF, 3TC, ATV/r</td><td>none</td><td>100</td></tr>
<tr><td>Y115F</td><td>ABC, 3TC, NVP</td><td>TDF, 3TC, EFV</td><td>6</td><td>50</td></tr>
<tr><td>L210W</td><td>AZT, 3TC, NVP</td><td>ABC, 3TC, LPV/r</td><td>none</td><td>21</td></tr>
</table>

L210W	AZT, 3TC, NVP	AZT, 3TC, LPV/r	none	27
T215Y	3TC, ABC, EFV	AZT, 3TC, EFV	none	95
T215Y	AZT, 3TC, NVP	ABC, 3TC, LPV/r	none	57
T215F	AZT, 3TC, NVP	ABC, 3TC, LPV/r	none	34
T215F	ABC, 3TC, NVP	TDF, 3TC, ATV/r	none	99
K219Q	AZT, 3TC, NVP	ABC, 3TC, LPV/r	none	99
K219Q	ABC, 3TC, NVP	TDF, 3TC, ATV/r	none	88
K219Q	ABC, 3TC, NVP	TDF, 3TC, EFV	none	99
K219Q	ABC, 3TC, NVP	TDF, 3TC, ATV/r	none	100

Footnote: 3TC, lamivudine; ABC, abacavir; AZT, zidovudine; D4T, stavudine; EFV, efavirenz; NRTI, nucleoside reverse transcriptase inhibitor; NVP, nevirapine; TP1, earlier time point; TP2, later time point.

Table S2. Evolving NNRTI DRMs

**Short-term evolution**				
DRM	Regimen		TP1 NGS frequency, %	TP2 NGS frequency, %
K103N	ABC, 3TC, EFV		none	99
K103N	ABC, 3TC, NVP		9	70
V108I	D4T, 3TC, NVP		none	34
Y181C	ABC, 3TC, NVP		10	96
Y181C	ABC, 3TC, NVP		16	49
H221Y	ABC, 3TC, NVP		none	87
H221Y	ABC, 3TC, NVP		none	41
F227I	D4T, 3TC, EFV		none	44
**Long-term evolution without regimen change**				
DRM	Regimen		TP1 NGS frequency, %	TP2 NGS frequency, %
Y181C	ABC, 3TC, NVP		3	85
Y181C	ABC, 3TC, NVP		10	99
H221Y	ABC, 3TC, NVP		none	39
H221Y	ABC, 3TC, NVP		7	99
**Long-term evolution with regimen change**				
DRM	TP1 regimen	TP2 regimen		
A98G	ABC, 3TC, EFV	AZT, 3TC, EFV	none	95
A98G	AZT, 3TC, NVP	ABC, 3TC, LPV/r	none	100
A98G	ABC, 3TC, NVP	TDF, 3TC, ATV/r	none	35
K101E	ABC, 3TC, NVP	TDF, 3TC, ATV/r	none	99
K103N	ABC, 3TC, NVP	AZT, 3TC, ATV/r	none	89
V106I	ABC, 3TC, NVP	AZT, 3TC, NVP	none	92

V108I	AZT, 3TC, NVP	ABC, 3TC, LPV/r	none	87
V108I	ABC, 3TC, NVP	TDF, 3TC, EFV	none	96
Y181C	D4T, 3TC, NVP	TDF, 3TC, ATV/r	15	92
H221Y	D4T, 3TC, NVP	TDF, 3TC, ATV/r	12	88
H221Y	ABC, 3TC, NVP	TDF, 3TC, ATV/r	none	87
H221Y	ABC, 3TC, NVP	TDF, 3TC, ATV/r	20	22
N348I	AZT, 3TC, NVP	ABC, 3TC, LPV/r	none	87
N348I	AZT, 3TC, NVP	AZT, 3TC, LPV/r	none	100

Footnote: 3TC, lamivudine; ABC, abacavir; AZT, zidovudine; D4T, stavudine; EFV, efavirenz; NNRTI, non-nucleoside reverse transcriptase inhibitor; NVP, nevirapine; TP1, earlier time point; TP2, later time point.

Table S3. Changes in NRTI DRM prevalence over time.

DRM	DRM evolution	Representative NGS threshold, %	Lower 95% CI	Difference, %	Upper 95% CI
L74V	Short Term	0	-0.2	2.6	21.7
L74V	Short Term	15	-41.8	36.2	75.6
L74V	Short Term	80	-13.4	67.8	96.1
L74V	Long Term	0	-0.2	2.9	33.6
L74V	Long Term	15	-3.5	32.5	90.6
L74V	Long Term	80	-13.0	70.8	95.9
Y115F	Short Term	0	0.4	2.7	11.1
Y115F	Short Term	15	-46.6	34.5	70.5
Y115F	Short Term	80	-10.7	87.0	95.3
Y115F	Long Term	0	0.4	4.0	65.3
Y115F	Long Term	15	-26.2	33.6	100.0
Y115F	Long Term	80	-11.1	84.8	100.0

Table S4. Changes in NNRTI DRM prevalence over time.

DRM	DRM evolution	Representative NGS threshold	Lower 95% CI	Difference	Upper 95% CI
A98G	Long Term	0	-96.2	-5.8	7.7
A98G	Long Term	15	-99.6	0.9	33.6
A98G	Long Term	80	-100.0	0.9	82.8
G190A	Long Term	0	-1.2	-0.2	0.0
G190A	Long Term	15	-66.3	-25.0	31.6

G190A	Long Term	80	-79.2	-24.3	81.1
H221Y	Long Term	0	-8.4	1.0	15.7
H221Y	Long Term	15	-28.1	14.1	66.4
H221Y	Long Term	80	-13.5	6.6	46.2
K103N	Long Term	0	-91.1	-8.3	1.3
K103N	Long Term	15	-76.3	-21.1	24.2
K103N	Long Term	80	-23.0	-1.7	29.6
V108I	Long Term	0	-4.4	0.2	6.6
V108I	Long Term	15	-99.4	2.6	98.7
V108I	Long Term	80	-99.9	1.2	99.2
Y181C	Long Term	0	-9.5	2.4	38.4
Y181C	Long Term	15	-9.7	19.3	66.7
Y181C	Long Term	80	-6.7	44.2	80.6

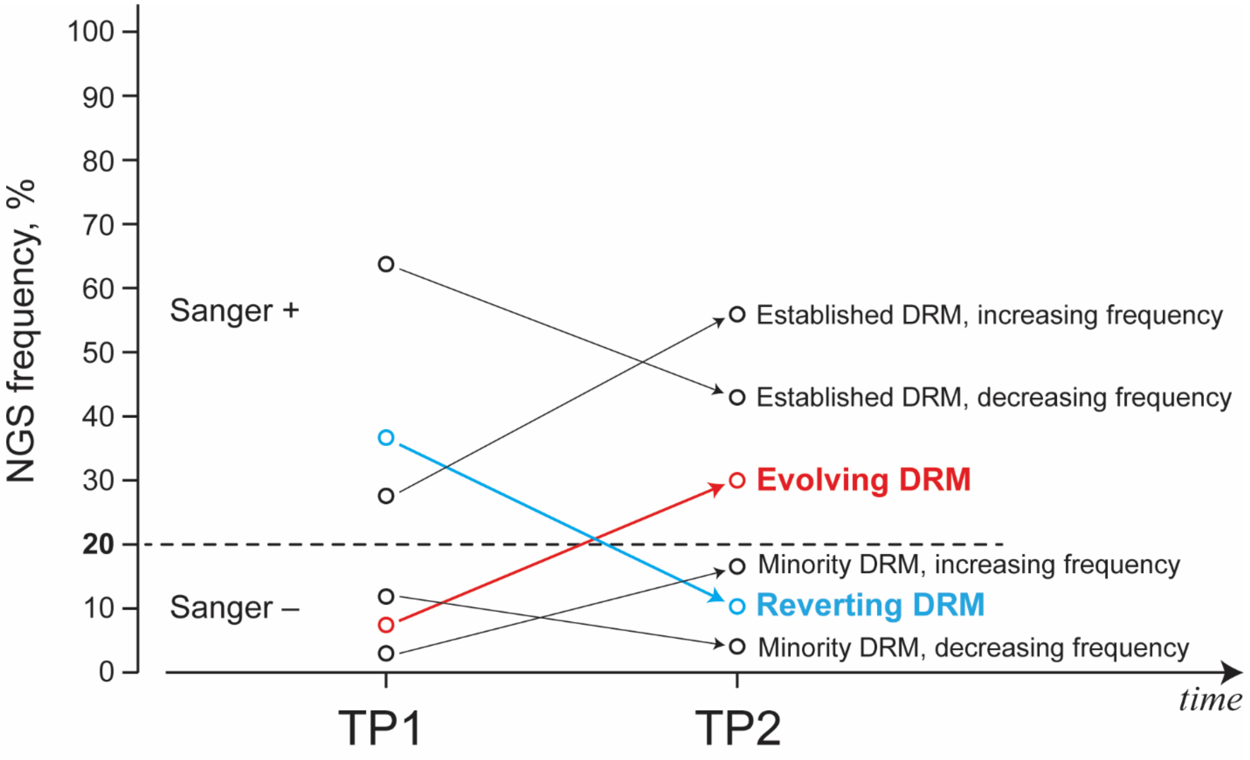

Supplementary Figure S1. Concept of DRM Evolution. This figure demonstrates schematically the different drug resistance evolution types described in this manuscript between an early and late TP (X axis), with hypothetical DRM NGS frequencies. The 20% mutation frequency threshold differentiating between minority and established DRM variants is indicated by the dashed line. DRMs above the 20% NGS threshold are usually detected by Sanger sequencing (Sanger+), while DRMs under the 20% threshold are not (Sanger–). DRMs that cross the 20% threshold between TPs are considered as evolving if increasing (red), or reverting if decreasing (blue). DRMs that remain above the 20% NGS threshold between TPs are defined as established with increasing or decreasing frequency, if frequency change is >5%; or as established stable, if change between TPs is within 5%. DRMs that remains below the 20% NGS threshold between TPs are defined as minority DRM with increasing or decreasing frequency, if frequency change is >5%; or as minority stable, if changes between TPs are within 5%. Stable DRMs (both established and minority) are not shown.

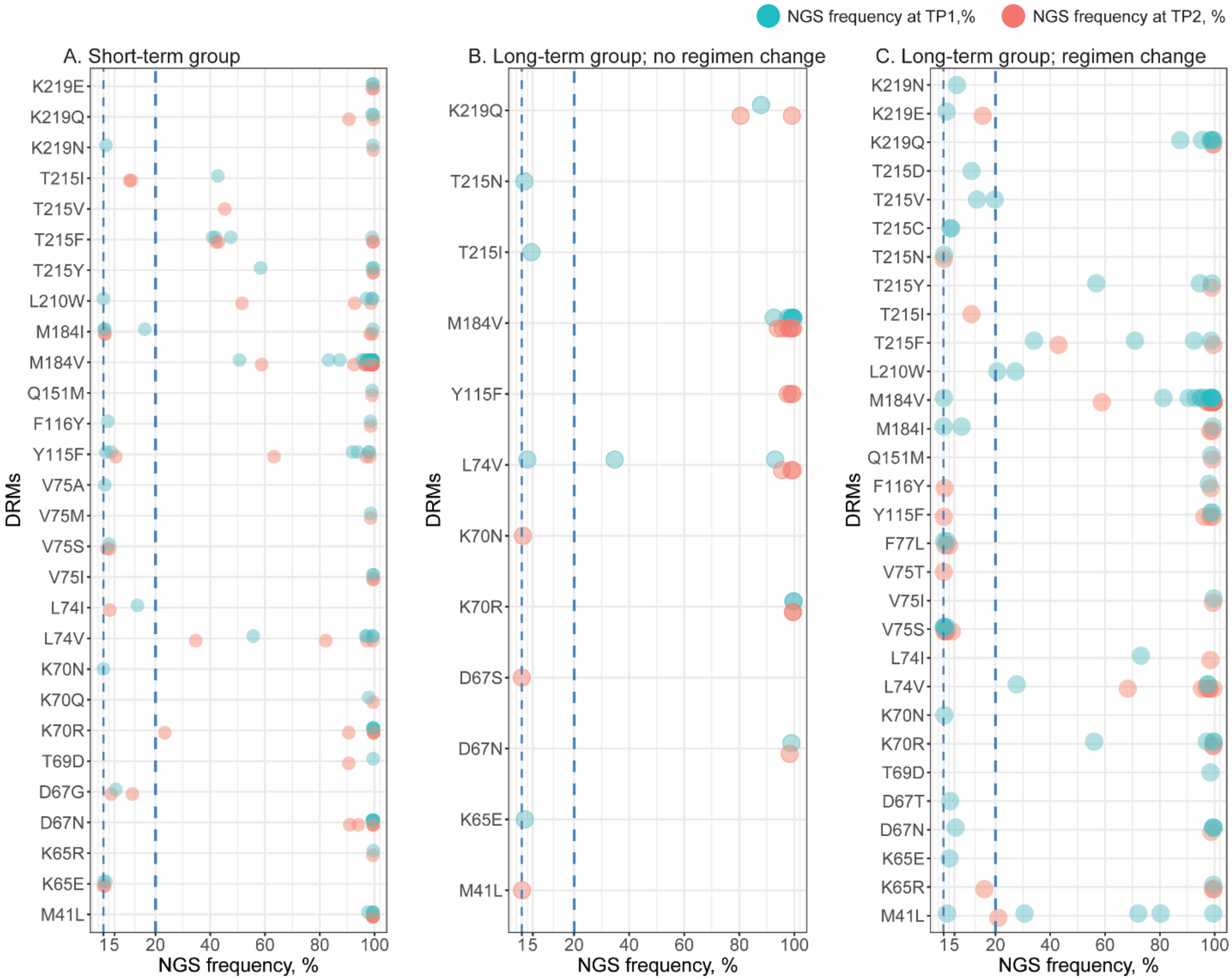

Supplementary Figure S2. Dynamics of NRTI DRMs. This figure demonstrates NGS frequencies (X axis) at earlier (blue dots) and later TPs (red dots) of NRTI DRMs (Y axis), identified in participants in the short-term (panel A), long-term without regimen change (panel B) and long-term with regimen change (panel C). Overlapping and partially overlapping dots indicate the same or similar NGS frequencies.

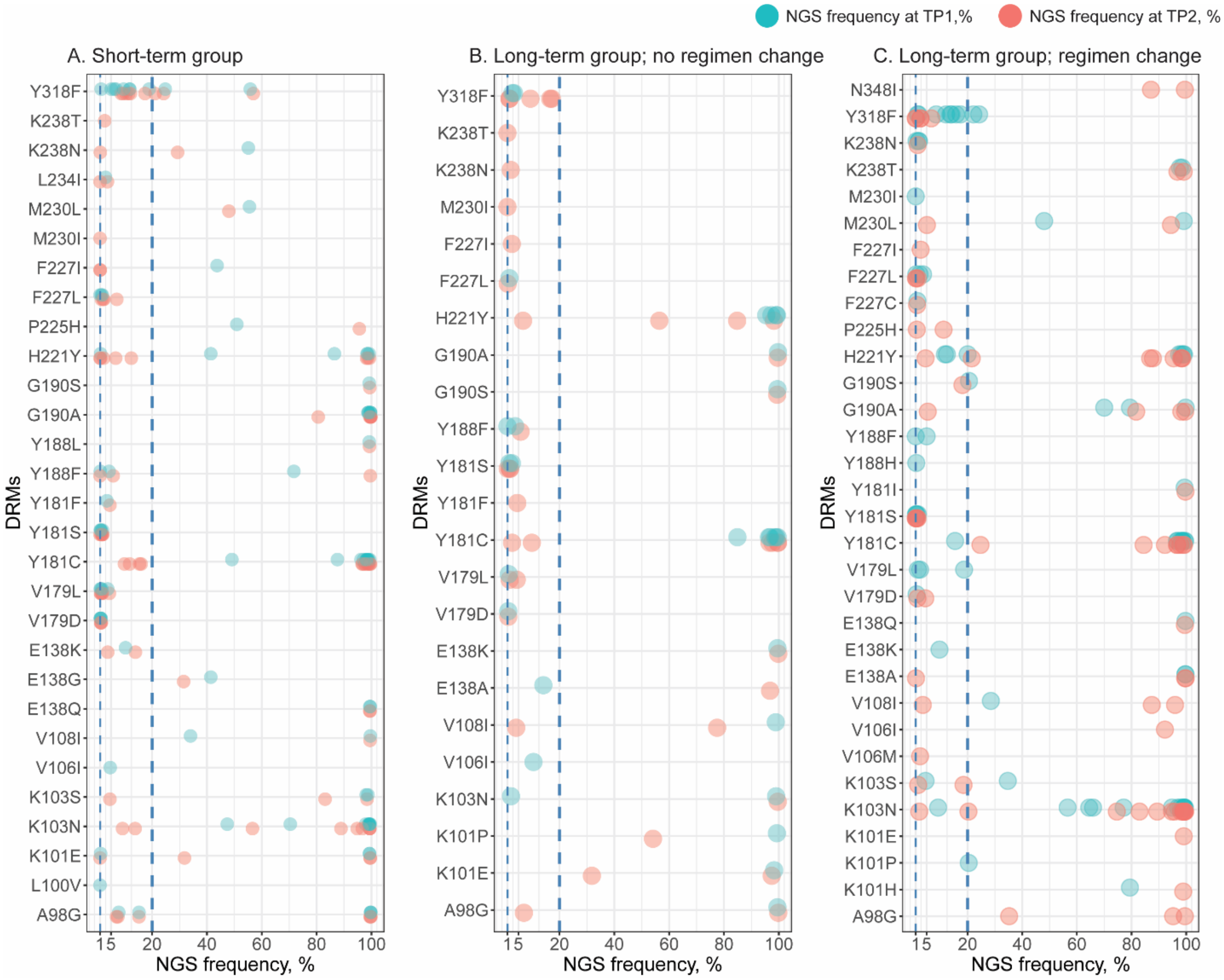

Supplementary Figure S3. Dynamics of NNRTI DRMs. This figure demonstrates NGS frequencies (X axis) at earlier (blue dots) and later TPs (red dots) of NNRTI DRMs (Y axis), identified in participants in the short-term (panel A), long-term without regimen change (panel B) and long-term with regimen change (panel C). Overlapping and partially overlapping dots indicate the same or close NGS frequencies.
